# FGFR Inhibitor AZD4547 Disrupts Inflammatory CAF Crosstalk With Cancer Cells and Macrophages and Attenuates Metastasis in Pancreatic Cancer

**DOI:** 10.1096/fj.202504573RR

**Published:** 2026-07-02

**Authors:** Ahmed M. R. H. Mostafa, Ahmed G. Hemdan, Franck Assayag, Jai Prakash

**Affiliations:** ^1^ Engineered Therapeutics, Department of Bioengineering Technology University of Twente Enschede the Netherlands; ^2^ Advanced Bioengineering and Therapeutics, Department of Medical Biosciences Radboud University Medical Centre Nijmegen the Netherlands; ^3^ Department of Pharmacology and Toxicology, Faculty of Pharmacy Assiut University Assiut Egypt

**Keywords:** fibroblast growth factor receptor, inflammatory cancer‐associated fibroblasts, liver metastasis, pancreatic ductal adenocarcinoma, tumor microenvironment, tumor stroma, tumor‐associated macrophages

## Abstract

Cancer‐associated fibroblasts (CAFs) are key cell types within the tumor microenvironment (TME), responsible for their pro‐tumorigenic effects and metastasis. Specifically, inflammatory CAFs (iCAFs), a CAF subtype, are known to induce tumor cell progression, migration, and immunosuppression. Fibroblast growth factor receptors (FGFRs) play a crucial role in cell differentiation, migration, and proliferation. In this study, we investigated the effect of FGFR kinase inhibitor AZD4547 (AZD), a clinical‐stage drug, on iCAF differentiation and iCAF‐mediated effects on the tumor‐stroma interaction in vitro and in vivo. Treatment with AZD inhibited the differentiation of human pancreatic stellate cells into iCAFs using IL‐1α, as shown with reduced IL‐6 expression. FGFR1, 2, 3, and 4 were upregulated in iCAFs, which were inhibited by AZD. Treatment with AZD also attenuated the iCAF‐mediated paracrine effect on the PDAC cell‐induced migration and epithelial‐mesenchymal transition of tumor cells, as well as polarization of macrophages towards the M1 phenotype in vitro. Furthermore, AZD significantly reduced the growth of tumor cells and fibroblasts in co‐cultured 3D heterospheroids in vitro. In vivo, treatment with AZD attenuated the tumor growth in the syngeneic subcutaneous KPC murine tumor model. Interestingly, flow cytometry and immunofluorescent staining analyses on isolated tumors revealed that AZD‐treated tumors had a reduced iCAF population and M2‐type macrophages. Furthermore, we found that AZD treatment reduced the liver metastasis, as shown with the reduction of ki‐67 and p53 tumor markers in the AZD‐treated group compared to the vehicle group. Altogether, this study demonstrates that FGFRs are overexpressed on iCAFs and their inhibition using AZD diminishes iCAF‐mediated paracrine signaling with tumor cells and macrophages, thereby attenuating tumor growth and metastasis.

## Graphical abstract

This study demonstrates that FGFR‐targeted inhibition by AZD4547 in inflammatory cancer‐associated fibroblasts (iCAFs) intervenes into the tumor–stroma crosstalk. AZD4547 blocks IL‐1α–induced differentiation of pancreatic stellate cells into iCAFs, impairing iCAF‐driven tumor cell differentiation, migration, and M2 macrophage polarization. Consequently, FGFR inhibition suppresses tumor growth and liver metastasis in the syngeneic KPC pancreatic ductal adenocarcinoma model in mice.

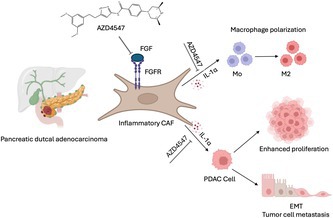

## 1 Introduction

The dynamic interplay between cancer cells and their surrounding tumor microenvironment (TME) plays a pivotal role in dictating the course of tumor progression and metastasis [1, 2]. Cancer‐associated fibroblasts (CAFs) are the prominent cell types within the TME which act as key regulators of the extracellular matrix (ECM) remodeling as well as cytokine production that drive tumor‐promoting programs [3, 4, 5]. In the past years, several subtypes of CAFs have been recognized, while myofibroblastic CAFs (myCAFs) and inflammatory CAFs (iCAFs) represent the most prominent subtypes in the tumor stroma of pancreatic ductal adenocarcinoma (PDAC) [6, 7]. MyCAFs, characterized by the abundant expression of alpha smooth muscle actin (α‐SMA), play a crucial role in ECM deposition, remodeling, and their contribution to the architecture of the TME [8, 9]. Conversely, iCAFs, while less involved in matrix remodeling [6], are more known for their secretome including IL‐6, IL‐11, CXCL12, CCL2, CCL22, and CCL1, thereby controlling the crosstalk with tumor cells as well as tumor immune cells [10, 11, 12].

The secretome of iCAFs can also play a pivotal role in promoting the epithelial‐mesenchymal transition (EMT) in tumor cells by secreting factors like IL‐6, thereby promoting tumor cells with enhanced migratory and invasive capabilities, along with an induced resistance to therapeutic interventions [13]. This transformative process augments the metastatic potential of tumor cells and reduces the therapeutic efficacy. Furthermore, this secretome is also known to actively skew the TME towards an immunosuppressive state, promoting the recruitment and activation of regulatory T cells, monocytes, and promoting their maturation towards myeloid‐derived suppressor cells, while simultaneously inhibiting cytotoxic CD8^+^ T cell infiltration and activity [12]. This suppression of the immune response actively allows tumor cells to escape immune surveillance and sets the stage for cancer progression [12]. With the mounting evidence of iCAF's role in cancer progression and metastasis, there is a need to develop strategies to modulate their pro‐tumoral function.

The fibroblast growth factor receptor (FGFR), a family of tyrosine kinase receptors, plays a significant role in various cellular processes, including proliferation, differentiation, and migration. FGFRs have been shown to be upregulated in different cancer types [14, 15, 16]. FGFRs are implicated in several key cellular processes, including proliferation, angiogenesis, and survival [17]. While in pancreatic cancer specifically, FGFR aberrations are increasingly recognized as drivers of tumor growth and progression, iCAFs, with their proteome's dual role in promoting immunosuppression and EMT, have emerged as prime targets [18, 19, 20, 21, 22].

AZD4547 (AZD, fexagratinib) is a clinical‐stage small‐molecule tyrosine kinase inhibitor that selectively targets FGFR1‐3 kinases, blocking FGFR‐mediated signaling, which plays a key role in tumor cell proliferation, survival, and angiogenesis [23]. AZD reduced the phosphorylation of signaling proteins involved in cell survival and apoptotic pathways, and its effects appeared to be mediated via Ras/MAPK and JAK/STAT pathways. In a phase IIa, multicenter, open label, single arm study (RADICAL; NCT01791985), AZD was administered with anastrozole or letrozole in estrogen receptor positive metastatic breast cancer patients who were resistant to aromatase inhibitors. 27% of patients discontinued treatment due to adverse effects, while others showed an overall response rate of 10%, meeting the prespecified endpoint [24]. Previous preclinical studies have also studied its effect on CAFs in general for their effect on CAF metabolism and mobility [25]. However, there are no studies showing the effect of AZD on myCAF and iCAF and in the context of pancreatic cancer.

In the present study, we aimed to assess the effect of AZD on iCAFs differentiation and iCAF‐mediated paracrine effects on tumor cells and macrophages. We first examined the expression of different FGF receptors on CAFs using publicly available single‐cell RNA sequencing (scRNAseq) data from PDAC patients. Then, we differentiated human pancreatic stellate cells (PSC) into iCAFs using IL‐1α and examined the expression of different FGFRs, followed by the effect of AZD on their differentiation in vitro. Thereafter, we examined the effect of AZD on iCAF‐mediated effect on human PANC‐1 cell migration and differentiation into mesenchymal phenotype. Furthermore, we developed a mouse 3D spheroid model by co‐culturing mouse KPC murine PDAC cells and NIH3T3 fibroblasts and examined the effect of AZD on the spheroid growth. Lastly, we evaluated the effect of AZD on tumor growth and liver metastasis. We further determined the effect on different CAFs and macrophage polarization in the KPC subcutaneous murine tumor model in vivo.

## 2 Materials and Methods

### 2.1 Cell Culture

Primary human pancreatic stellate cells (PSCs), a product of ScienCell Research Laboratories, were purchased from Sanbio B.V., Uden, Netherlands and cultured in complete stellate cell medium (SteCM; Sanbio), supplemented with 2% fetal bovine serum (FBS; Sanbio), 1% medium‐specific stellate cell growth supplement (SteCGS) and 1% penicillin/streptomycin antibiotic supplement (Sanbio). Human PSCs were cultured up to passage 10. Human pancreatic tumor cell lines, PANC‐1 and Murine NIH3T3 embryo fibroblasts, were purchased from American Type Culture Collection (ATCC; Virginia) and cultured in high glucose: 4.5 g/L Lonza Dulbecco's Modified Eagle Medium (DMEM), without l‐glutamine (Westburg B.V., Leusden, Netherlands), supplemented with 10% fetal bovine serum (FBS; Sigma‐Aldrich), 2 mM l‐glutamine and 1% penicillin/streptomycin (Sigma‐Aldrich). THP‐1 monocyte cell line was purchased from ATCC and maintained in ATCC‐modified Gibco RPMI 1640 Medium (ThermoFisher Scientific), supplemented with 10% in‐house heat‐inactivated FBS (Sigma‐Aldrich) and 1% penicillin/streptomycin. Luciferase‐expressing LSL‐Kras^G12D/+^; LSL‐Trp53^R172H/+^;Pdx‐1‐Cre (KPC3‐Luc2) murine pancreatic tumor cells were gifted by Dr. Lukas Hawinkels, Leiden University Medical Center, Netherlands, and cultured in Gibco Iscove's Modified Dulbecco's Medium (IMDM; ThermoFisher) growth medium, supplemented with 8% FBS, 2 mM l‐glutamine and 1% penicillin/streptomycin. Cells were maintained at 37°C with 5% CO_2_ under humidified conditions.

### 2.2 Immunocytochemistry Staining

In order to investigate the effect of AZD (Fexagratinib, ADSK091; Tebu‐bio B.V., Heerhugowaard, Netherlands) on the activation state of iCAFs, intracellular IL‐6 was employed as an iCAF marker. 1 × 10^4^ human PSC cells were seeded into a 24‐well‐plate. After overnight incubation, cells were starved for 24 h before being activated with 1 ng/mL of human IL‐1α (Peprotech, London, UK) with or without 0.5 μM AZD treatment. 24 h post‐treatment, cells were fixed with 4% formaldehyde (Sigma‐aldrich, Amsterdam, Netherlands) for 10 min at room temperature for further immunostaining procedure. Cells were permeabilized using 0.1% of Triton X‐100 (Sigma‐aldrich) in 2% bovine serum albumin (BSA; VWR B.V., Amsterdam, Netherlands) blocking buffer for 1 h in room temperature. Subsequently, the fixed cells were incubated overnight at 4°C with primary antibodies (see details in Table S2). Next day, the cells were incubated for 1 h at room temperature with the corresponding Alexa Fluor‐conjugated secondary antibodies (Table S2). Cells were then mounted with Fluoroshield containing DAPI (Sigma‐Aldrich) for nuclear staining. Images were acquired using an EVOS FI fluorescence microscope (Life Technologies, Bleiswijk, The Netherlands) at excitation/emission wavelengths of 357/447 nm for DAPI, 482/524 nm for the green channel, and 585/628 nm for the red channel.

### 2.3 Gene Expression

Gene expression levels were quantitatively assessed using Quantitative Reverse Transcription Polymerase Chain Reaction (RT‐qPCR). The process began with seeding human PSCs in 12‐well plates at a density of 40, 000 cells per well. After 24 h, which allowed the cells to adhere and recuperate, they were exposed to serum‐free media for another 24 h to induce starvation. Following this, the cells were treated with human IL‐1α (1 ng/mL; Peprotech), with and without AZD (0.5 μM), for 24 h. Cell lysates were then obtained using a lysis buffer that included 2‐Mercaptoethanol (Sigma‐Aldrich), and total RNA was extracted using the GenElute Mammalian Total RNA Isolation Kit (Sigma‐Aldrich). RNA concentration was measured via spectrophotometry with the Nanodrop UV–Vis Spectrometer (Thermo Fisher Scientific). RNA was then converted to complementary DNA (cDNA) using the iScript cDNA Synthesis Kit (Bio‐Rad Laboratories B.V., Veenedaal, Netherlands) and processed in a thermocycler set to a pre‐determined program.

For the RT‐qPCR analysis, 4 μg of cDNA was used together with specific forward and reverse primers and the Bioline SYBR Green mix (Meridian Bioscience, Memphis, Tennessee). Details of the primers used are provided in Table S1.

### 2.4 Conditioned Media Generation

In order to test CAF‐derived paracrine effect on tumor cells and macrophages, conditioned media are collected from treated CAFs. human PSCs are seeded in a total density of 4 × 10^4^ cells per well in 12 well‐plate. Cells are subsequently starved, IL‐1α activated and treated as previously described. 24 h post‐treatment, cells were washed 3× using starvation medium and incubated with fresh starvation medium for another 24 h. In case of the AZD treatment, PSCs were treated with IL‐1α and AZD for 24 h and then washed 3× to remove any IL‐1α and AZD. Later, conditioned medium was collected and decellularized by centrifugation at 500 *g* for 5 min and the supernatant is stored at −80°C for further studies.

### 2.5 Cell Viability Studies

In order to evaluate the effect of CAF‐derived conditioned medium on tumor cell growth, human pancreatic tumor cell lines; PANC‐1 cells were seeded into a 96 well plate at 0.5 × 10^4^ of total cell density per well. After an overnight incubation, the cells were serum‐starved and incubated with 100 μL of different CAF‐derived conditioned media on the next day. After 72 h of conditioned media incubation, cells were incubated with 1× resazurin‐based (Alamar Blue; Sigma‐aldrich) fluorescent dye for 4 h to assess mitochondrial activity. Fluorescence intensity was measured using a Victor plate reader (Perkin Elmer, Waltham, Massachusetts) and detected at ex./em. 540/590 nm.

### 2.6 Transwell Migration Assay

Transwell migration was used to aid in evaluating the chemotaxis effect of CAF‐derived paracrine signaling on PANC‐1 tumor cells migration through an 8 μm chamber membrane. Briefly, PANC‐1 cells were seeded into the upper chamber of 24 well‐plate transwell inserts (Sigma‐aldrich) at a total density of 5 × 10^4^ cells per insert, with underlying CAF‐derived conditioned media in the lower chambers. 24 h later, transwell inserts were collected and incubated with ice‐cold methanol (Honeywell B.V., Delft, Netherlands) in −20°C for 10 min in order to fix the cells. Next, transwell membrane‐attached cells were stained by incubation with 0.1% Crystal Violet (Sigma‐aldrich) in 25% methanol solution for 10 min at room temperature. Excess Crystal Violet was eliminated by washing the inserts with Milli‐Q water and cells attached to the upper compartment were mechanically removed by cotton swab. Migrated, Crystal Violet‐stained PANC‐1 cells were visualized at the lower compartment side using Nikon Eclipse E400 microscope (Nikon, Tokyo, Japan).

### 2.7 Macrophage Activation

In order to evaluate the effect of CAF‐derived paracrine signaling on macrophage polarization and activation, THP‐1 cells were employed as a macrophage progenitor model. THP‐1 cells were treated with 100 nM of Phorbol 12‐myristate 13‐acetate (PMA; Invivogen, California) and seeded at a density of 30 × 10^4^ cells per well in 12 well‐plate. After overnight incubation to attach, macrophage polarized cells were washed 3× and incubated with fresh complete medium for 24 h to rest. On the next day, cells were incubated either with M1‐polarizing cytokines mixture (20 ng/mL IFN and LPS), M2‐polarizing cytokine mixture (20 ng/mL IL‐4 and 20 ng/mL IL‐13; Peprotech) or CAF‐derived conditioned medium, supplemented with 10% heat‐inactivated FBS, 10 mM 4‐(2‐hydroxyethyl)‐1‐piperazineethanesulfonic acid (Gibco HEPES; ThermoFisher Scientific) and 1 mM sodium pyruvate (ThermoFisher Scientific). After 72 h of incubation, cells were washed and detached using a PBS buffer mixture of 1% Bovine serum albumin (BSA; VWR), 0.1% sodium azide (Sigma‐aldrich) and 5 mM sodium Ethylenediaminetetraacetic acid (EDTA; Sigma‐aldrich). Next, detached cells were counted and centrifuged at 120 *g* for 5 min and resuspended in FACS buffer, consisting of 1% BSA, 0.1% sodium azide and 2 mM EDTA. Cells were blocked for 10 min in Fc Receptor Binding Inhibitor Polyclonal Antibody (ThermoFisher Scientific) and incubated with labeled anti‐human CD86 and anti‐human CD206 antibodies (BioLegend, San Diego, California) for 1 h at 4°C. Subsequently, cells were 2× washed in FACS buffer and flow cytometry analysis was carried for at least 1 × 10^4^ gated events using MACSQuant Analyzer (Miltenyi Biotec, Bergisch Gladbach, Germany). Data were analyzed using Floreada.

### 2.8 3D Tumor Spheroid Growth Assay

In order to predict the effect of AZD on tumor growth in vivo, recapitulating, in vitro stroma‐containing 3D tumor models were employed. Round‐bottom 96‐well plates were initially prepared by precoating them with a 1% (w/v) solution of Poloxamer 407 (Pluronic F‐127; ThermoFisher Scientific). Following an overnight incubation, these plates were washed three times using sterile Milli‐Q water to ensure optimal surface preparation. To generate KPC/3T3 3D spheroids, cells were seeded at an overall density of 1.2 × 10^4^ cells/well at a cell ratio of 1:5, respectively in 100 μL. This included both KPC monoculture tumor homospheroids and combined KPC/3T3 stroma‐rich heterospheroids. Culture medium was composed of complete DMEM medium. In parallel, to form PANC‐1/PSCs 3D spheroids, cells were introduced at a density of 0.6 × 10^4^ cells/well at a cell ratio of 1:5, respectively. The medium employed for this purpose was a balanced 1:1 (v/v) mixture of complete DMEM and stellate cell medium. Within a span of 72 h, spheroids were spontaneously formed and treated with AZD. Preliminary images as well as on day 6, 9 and 12 were captured using EVOS microscope in order to track spheroids volume growth. Subsequently, volume of spheroids was determined using every time point measured areas according to the formula: $\text{Volume}=\frac{4}{3}\times \pi \times {\text{radius}}^{3}$.

### 2.9 Fluorescent Labelling of KPC3‐Luc2 and NIH3T3

In order to determine the intra‐spheroid cellular arrangement after spheroid formation, KPC3‐Luc2 and NIH3T3 were fluorescently labeled with different fluorescent dyes. KPC3‐Luc2 cells were incubated with CellTracker Red CMTPX dye (ThermoFisher Scientific) and NIH3T3 fibroblasts were incubated with CellTracker Green CMFDA (ThermoFisher Scientific) for 30 min at 37°C prior to cells detachment and spheroid formation. 72 h later, spheroids were spontaneously formed and subsequently Cryomatrix‐embedded and snap‐frozen using ice‐cold isopentane (Sigma‐aldrich). Cryofrozen spheroids were cut into 6 μm‐thick cryosections and fixed using 4% formaldehyde for 10 min at room temperature in the dark. Later, fixed tissues were mounted using Fluoroshield with DAPI for fluorescence microscopy imaging using EVOS, and signals were detected at ex./em. 357/447 nm for DAPI, 482/524 nm for the green channel, and 585/628 nm for the red channel.

### 2.10 Spheroids Topography

In order to visualize the spheroids morphology, spheroids were fixed using 4% formaldehyde for 30 min at room temperature in a dark environment and subsequently washed with Milli‐Q water. That was followed by cryofreezing spheroids suspension in liquid nitrogen and freeze‐dried. Freeze‐dried spheroids were later mounted on specimen stubs and gold sputter‐coated under Argon purge (Sputter coater 108 Auto; Cressington Scientific Instruments, Watford, UK). Later, spheroids were visualized using a JEOL scanning electron microscope (SEM; JSM‐IT100; JEOL Ltd., Tokyo, Japan).

### 2.11 In Vivo Animal Study

The experimental working protocol was ethically approved by the institutional animal welfare body, University of Twente and the Central Commission for Animal Experiments of the Netherlands and carried out under project license number: AVD1100020174305, WP2022‐4305‐JP03. 8‐week‐old female C57BL/6JRj mice were purchased from Charles River Laboratories (Gottingen, Germany). Animals were housed in climate‐controlled Individually Ventilated Cages (IVC) housing under a temperature of 20°C–24°C and humidity of 40%–70% with a regular 12 h light/12 h dark cycle. After 1 week of acclimatization, tumor challenge was initiated via the inoculation of 1 × 10^5^ KPC3‐Luc2 cells in 100 μL (0.1% BSA in PBS) subcutaneously in the mice right flank area under general inhalation anesthesia using 3% isoflurane for induction and 1.5% maintenance in order to generate KPC pancreatic ductal adenocarcinoma (PDAC) tumor model. Approximately, 2 weeks later, as soon as tumor volumes reach 50–100 mm^3^ of volume, mice were randomly allocated to respective treatment groups and intraperitoneally injected with either PBS or AZD (10 mg/kg/day) 3× per week for 2 weeks based on a previous study [26]. Tumor volumes and body weights were recorded 3× per week until the humane endpoint (1 × 10^3^ mm^3^ of tumor volume or loss of body weight > 15% within the previous 48 h). Mice were intraperitoneally injected with d‐Luciferin (100 mg/mL; MedChemExpress) 15 min prior to euthanasia. Tumor and major organs were collected, weighed, and imaged for bioluminescence using the Pearl Trilogy animal imaging system (LI‐COR Biosciences, Lincoln, Nebraska). Subsequently, part of the tumor was digested for further flow cytometry analysis, and the rest of the tumor and organs were preserved via Epredia Cryomatrix resin (ThermoFisher Scientific)‐embedding and snap‐frozen in ice‐cold Isopentane (Sigma‐Aldrich) for further histological analysis.

### 2.12 Flow Cytometry Analysis

Parts of tumor tissues were minced into small pieces using scalpel and enzymatically digested for later flow cytometry analysis by incubation in a digestion buffer of DMEM medium consisting of 400 U/mL of collagenase type II (Sigma‐aldrich), 200 U/mL of Deoxyribonuclease I (DNAse; Sigma‐Aldrich), 0.25 U/mL Dispase II (Sigma‐aldrich) and supplemented with 20 mM of HEPES buffer for 2 h in 37°C under mild agitation. Subsequently, digested tumor fragments were passed through 70 μm cell strainers (VWR), in order to exclude undigested fragments, centrifuged and PBS washed for 5 min at 300 *g*. After cell counting, single‐cell suspensions were fixed for 10 min in 4% formaldehyde and washed with PBS. Next, fixed cell suspensions were permeabilized using 0.1% Triton X‐100 for 15 min at room temperature, washed and incubated with primary antibodies for 1 h, followed by incubation with species‐specific corresponding Alexa Fluor‐labeled (ThermoFisher Scientific) secondary antibodies for 1 h at RT. After washing and resuspension, samples analysis was run on MACSQuant flow cytometer (Miltenyi Biotec, Gladbach, Germany).

### 2.13 Immunofluorescence Staining

Frozen Cryomatrix resin‐embedded tumors were sectioned using cryostat (MNT; SLEE Medical, VWR) into 8 μm‐thick cryosections, dried under hair‐dryer at room temperature and fixed using 100% acetone (VWR) for 10 min at room temperature. Eventually, fixed tissues were delineated using hydrophobic pen, followed by blocking using blocking solution of 3% BSA buffered in PBS. Immunohistochemistry staining was initiated by incubating the sections with primary antibodies in blocking solution overnight at 4°C. That was followed by incubation with the corresponding species‐specific Alexa Fluor‐conjugated secondary antibodies for 1 h at room temperature (Table S2). Subsequently, samples were mounted using Fluoroshield containing DAPI for nuclear staining. After overnight curing at 4°C, slides were imaged for fluorescence using the NanoZoomer Digital Slide Scanner 2.0HT (Hamamatsu Photonics, Hamamatsu, Japan).

### 2.14 Immunohistochemical Staining

Isolated livers were sectioned (8 μm‐thick) and followed by drying. Fixation was obtained via 4% formaldehyde incubation for 15 min at room temperature. 0.3% hydrogen peroxide solution (ThermoFisher) in methanol was used for 30 min to block endogenous peroxidase activity. Following a rinse with MilliQ water, the samples were permeabilized for nuclear staining using 0.1% Triton X‐100 (Sigma‐Aldrich) in 2% BSA for 30 min. Next, sections were incubated with primary antibody against Ki‐67 (NBP2‐547911:200; Novous Biologicals), p53 (MA5‐12453, ThermoFisher) diluted in 2% BSA in PBS at 4°C overnight. Following a subsequent PBS wash, the sections were incubated with species‐specific HRP‐conjugated secondary and tertiary antibodies (1:100; Agilent Technologies Netherlands B.V., Middelburg, The Netherlands), diluted in 2% BSA in PBS, for 1 h at room temperature. The visualization of HRP‐conjugated antibodies was achieved with a 3‐Amino‐9‐ethylcarbazole (AEC; Agilent Technologies) solution for 5–10 min, followed by a 2‐min hematoxylin counterstaining. The sections were then dehydrated and mounted using VectaMount (Vector Laboratories, California). Imaging of all stained sections was conducted using a Hamamatsu NanoZoomer whole slide scanner.

## 3 Results

### 3.1 FGF Receptors Expression in PDAC and Fibroblasts Subtypes

**FIGURE 1:**
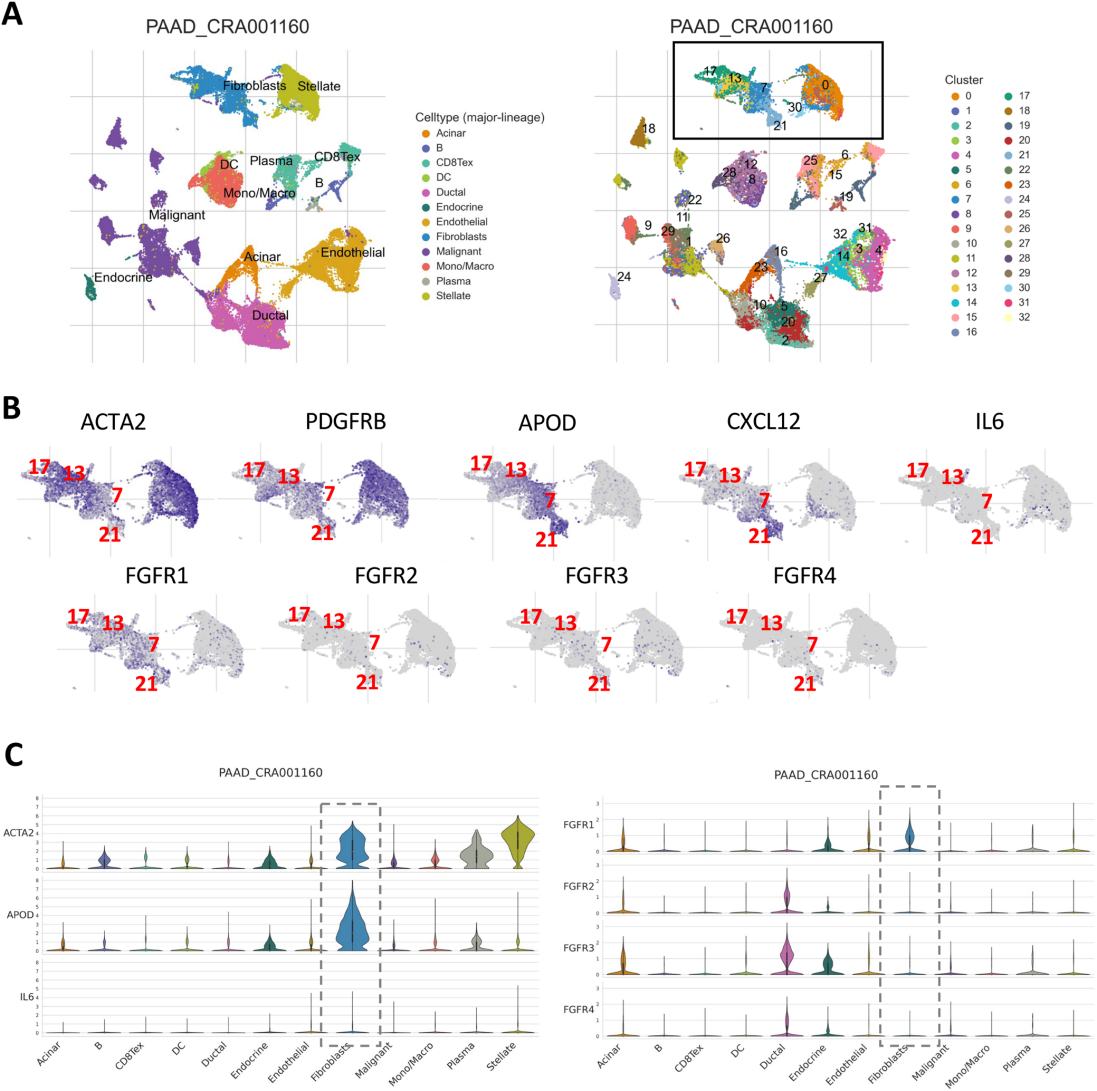
Expression of different FGF receptors in human PDAC and iCAFs. (A) Publicly available scRNAseq data from PDAC patients showing different cell types in the tumor (analyzed using tisch.compbio.cn). Each cell type is subdivided into 32 clusters; especially fibroblasts are clustered in 7, 13, 17, 21, 30, and pancreatic stellate cells as 0. (B) Based on biomarkers (*ACTA2, PDGFRB*), cluster 17, 13, and 30 are recognized as myCAF, while based on biomarkers (*APOD, CXCL12, IL6*), cluster 7 and 21 were recognized as iCAF. (C) Expression of *ACTA2, APOD*, and IL6 and different FGFRs in various cells within PDAC scRNAseq data. Dotted box represents expression in fibroblasts. FGFR1 expression is the most expressed receptor in fibroblasts and is spread over all clusters, while other FGF receptors were weakly expressed.

Using publicly available scRNAseq data in clinical PDAC samples [27], we tried to delineate the expression of different FGF receptors using an online platform which shows heterogenous cell types and their clusters for different phenotypes (Figure 1A). Among different FGF receptors, *FGFR1* was the main receptor overexpressed in the fibroblast clusters and weakly in the stellate cell cluster, while other FGF receptors show a weak expression in the fibroblast clusters but in ductal and endothelial cell clusters (Figure S1). The fibroblast cluster is subdivided into 4 clusters (cluster 7, 13, 17, and 21). Based on the gene markers for myCAF and iCAF, we found that clusters 13 and 17 presented largely myCAF as they overexpressed *ACTA2* and platelet‐derived growth factor receptor beta (*PDGFBR*), while clusters 7 and 21 largely present iCAF due to overexpression of apolipoprotein D (*APOD*), *CXCL12* and *IL6* (iCAF markers [7]) (Figure 1B). Furthermore, *FGFR1* was found to be expressed in different fibroblast clusters in the following order: cluster#21 > cluster#13 > cluster#7 > cluster#17, indicating that this receptor is overexpressed by *CXCL12* ^+^ iCAF in comparison to *ACTA2* ^+^ myCAF (Figure 1C).

### 3.2 AZD Inhibits FGFR Expression and iCAF Differentiation In Vitro

**FIGURE 2:**
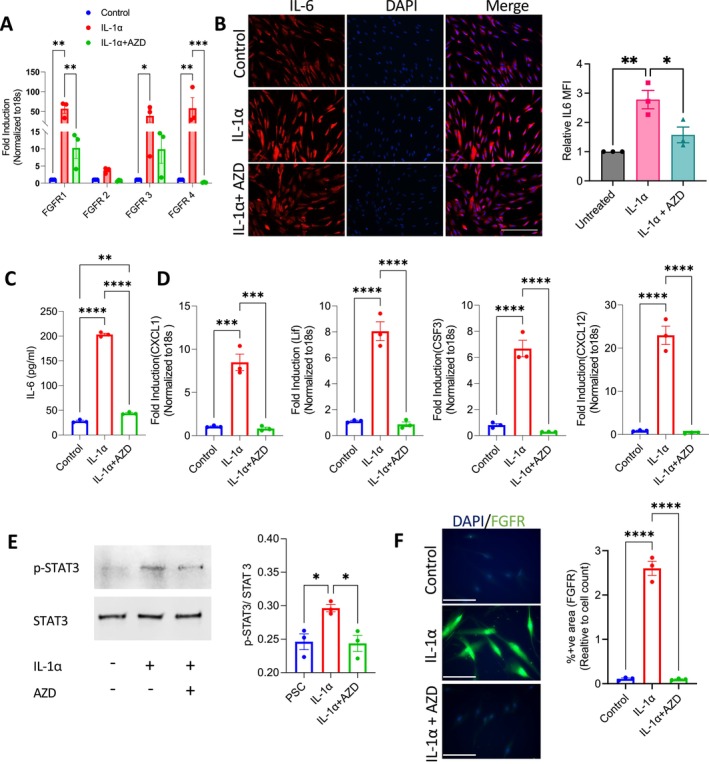
AZD4547 inhibits iCAF activation and suppresses FGFR signaling in vitro. (A) Quantitative PCR analysis demonstrating the effect of AZD4547 on FGFR1–4 gene expression in IL‐1α–differentiated human PSCs. Expression levels were normalized to the housekeeping gene *RPS18*. Data are presented as mean ± SEM (*n* = 3) and analyzed using two‐way ANOVA (**p < 0.01, ***p < 0.001). (B) Immunocytochemical analysis of FGFR1 expression in IL‐1α–differentiated human PSCs. Data are presented as mean ± SEM (*n* = 3), and statistical comparisons were performed using one‐way ANOVA (****p < 0.0001). Scale bar: 200 μm. (C) Representative fluorescence microscopy images and quantification of immunofluorescence staining showing intracellular IL‐6 expression in human PSCs with or without AZD4547 treatment. Scale bar: 200 μm. (D) ELISA assay demonstrating that AZD4547 significantly reduces IL‐6 secretion in IL‐1α–activated PSCs. Data are presented as mean ± SEM from 3 independent experiments and analyzed using one‐way ANOVA (**p < 0.01, ****p < 0.0001). (E) Quantitative PCR analysis showing downregulation of key iCAF‐associated genes, including *CXCL1, CXCL3, LIF*, and *CXCL12*, following AZD4547 treatment. Data are presented as mean ± SEM from three independent experiments and analyzed using one‐way ANOVA (**p < 0.01, ***p < 0.001, ****p < 0.0001). (F) Western blot analysis (representative blots and densitometric quantification) showing that AZD4547 inhibits STAT3 phosphorylation (p‐STAT3), indicating suppression of downstream FGFR signaling. Data are presented as mean ± SEM from three independent experiments and analyzed using one‐way ANOVA (*p < 0.05).

To examine the effect of AZD on the expression levels of different FGF receptors, we generated iCAFs by differentiating human PSCs in the presence of IL‐1α in vitro. Analysis of FGF receptor expression revealed that all four FGFRs (FGFR1–4) were upregulated in iCAFs (Figure 2A). Notably, *FGFR1* and *FGFR3* exhibited markedly higher expression levels (> 60‐fold increase), whereas *FGFR2* and *FGFR4* showed a more modest induction (~8‐fold increase). In comparison, our previous work demonstrated that in myCAFs, only FGFR3 was modestly upregulated (~2‐fold) [16]. We found that treatment with AZD reduced the expression of all of these receptors significantly (Figure 2A). Consistent with the gene expression data, immunocytochemical analysis revealed a marked increase in FGFR1 protein expression upon IL‐1α–induced differentiation, which was substantially reduced following AZD treatment (Figure 2B). Furthermore, treatment with AZD at a concentration of 0.5 μM significantly reduced the expression levels of IL‐6, as shown with immunofluorescence staining analysis (Figure 2C) and markedly decreased IL‐6 secretion, as quantified by ELISA (Figure 2D). Furthermore, AZD treatment significantly suppressed the expression of key iCAF‐associated genes, including *CXCL1*, *CXCL12*, leukemia inhibitory factor (*LIF*), and colony stimulating factor 3 (*CSF3*) (Figure 2E). To explore the underlying mechanism, western blot analysis revealed that AZD inhibited IL‐1α‐induced STAT3 phosphorylation (Figure 2F), suggesting that AZD modulates iCAF activation through suppression of the IL‐1α/STAT3 signaling axis.

Our results demonstrate the pivotal role of FGFRs inhibition on iCAFs, specifically via AZD in dictating the human PSCs differentiation towards iCAFs. When we tested the effects of AZD on TGF‐β‐activated PSCs into myCAFs, we found that there was a further induction of α‐SMA and collagen expressions (Figure S1). These effects were in agreement with our previous study in which recombinant FGF inhibited the myCAF differentiation [28] and inhibition of FGFR by AZD, as shown in this study, maintained the activated myCAFs phenotype.

### 3.3 AZD Inhibits iCAF‐Induced Paracrine Signaling in Tumor Cell Migration and EMT

**FIGURE 3:**
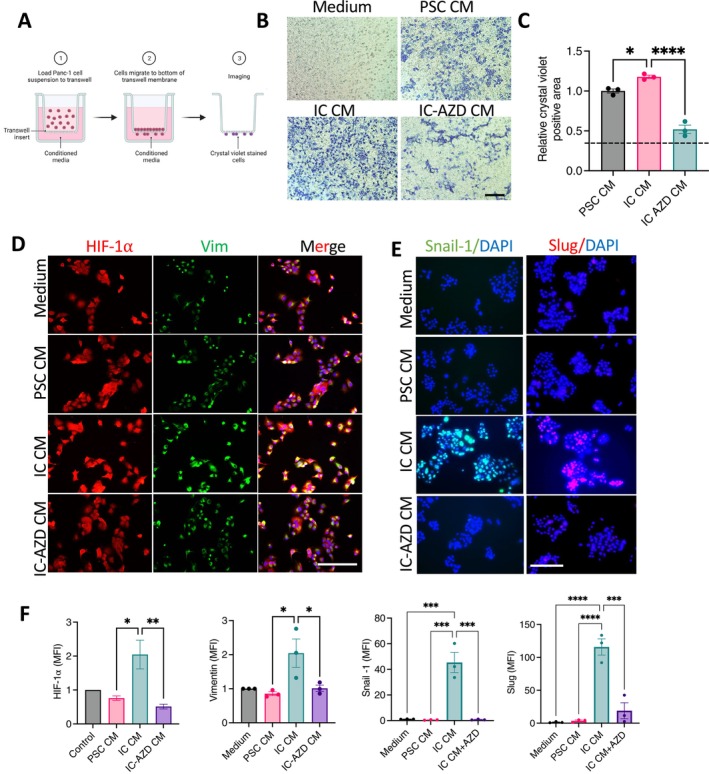
AZD4547 suppresses iCAF‐driven epithelial–mesenchymal transition (EMT) in tumor cells. (A) Schematic illustration of transwell migration assay. (B) Representative microscopy images and (C) quantification of crystal violet–stained migrated Panc‐1 cells in the lower chamber following treatment with different conditioned media. (D, E) Representative immunofluorescence images showing expression of EMT‐associated markers in Panc‐1 cells after 48 h of incubation with indicated CM treatments, including co‐staining of vimentin with HIF‐1α, Vimentin, Snail‐1, and Slug. (F) Corresponding quantitative analysis of fluorescence intensity for EMT marker expression. All data are presented as mean ± SEM (*n* = 3). Scale bar: 200 μm. Statistical significance was determined using one‐way ANOVA (*p < 0.05, **p < 0.01, ***p < 0.001, ****p < 0.0001).

After validating the effect of AZD on iCAF differentiation, we aimed to elucidate the direct effects of iCAFs on tumor cell migration and examine the potential inhibitory effect of AZD on this crosstalk. To accomplish this, we collected conditioned medium (CM) from iCAFs and applied it to tumor cells to study the paracrine effect on migration and EMT. To further investigate the role of iCAFs in tumor cell migration, we performed a transwell insert migration assay using human PANC‐1 tumor cells (Figure 3A). These data revealed that there was a pronounced increase in PANC‐1 migration upon exposure to CM derived from human PSC and even more when exposed to iCAFs‐CM in a chemotactic migratory manner. Of note, this enhanced migratory behavior was significantly inhibited when the iCAF‐CM was derived from AZD‐treated iCAFs (Figure 3B,C), indicating that AZD inhibited iCAF differentiation and thereby potentially the secretion of chemotactic cytokines. In addition, we investigated the effect of AZD on the proliferation of both control and IL‐1α–treated hPSCs and found a slight reduction in proliferation in both conditions. This confirms that the changes observed in migratory behavior of iCAF‐conditioned media exposure are independent of any proliferative effects of AZD (Figure S2).

To further support our findings, we investigated whether iCAFs promote EMT in tumor cells. Given our observation that AZD suppresses IL‐1α‐induced STAT3 activation in iCAFs, we next examined whether this signaling axis contributes to EMT induction in pancreatic cancer cells. We therefore assessed the expression of key EMT markers, including vimentin, Snail1, and Snail2 (Slug). Notably, PANC‐1 cells cultured with iCAF‐CM exhibited a marked upregulation of vimentin, Snail1, and Slug, along with increased expression of HIF‐1α, a hypoxia‐associated marker (Figure 3D–F). In contrast, this effect was significantly attenuated when cells were exposed to CM from AZD‐treated iCAFs, consistent with inhibition of IL‐1α/STAT3‐driven iCAF activation. Collectively, these findings suggest that AZD suppresses iCAF‐mediated paracrine induction of EMT in tumor cells.

### 3.4 AZD Attenuates iCAF‐Induced M2 Macrophage Polarization

**FIGURE 4:**
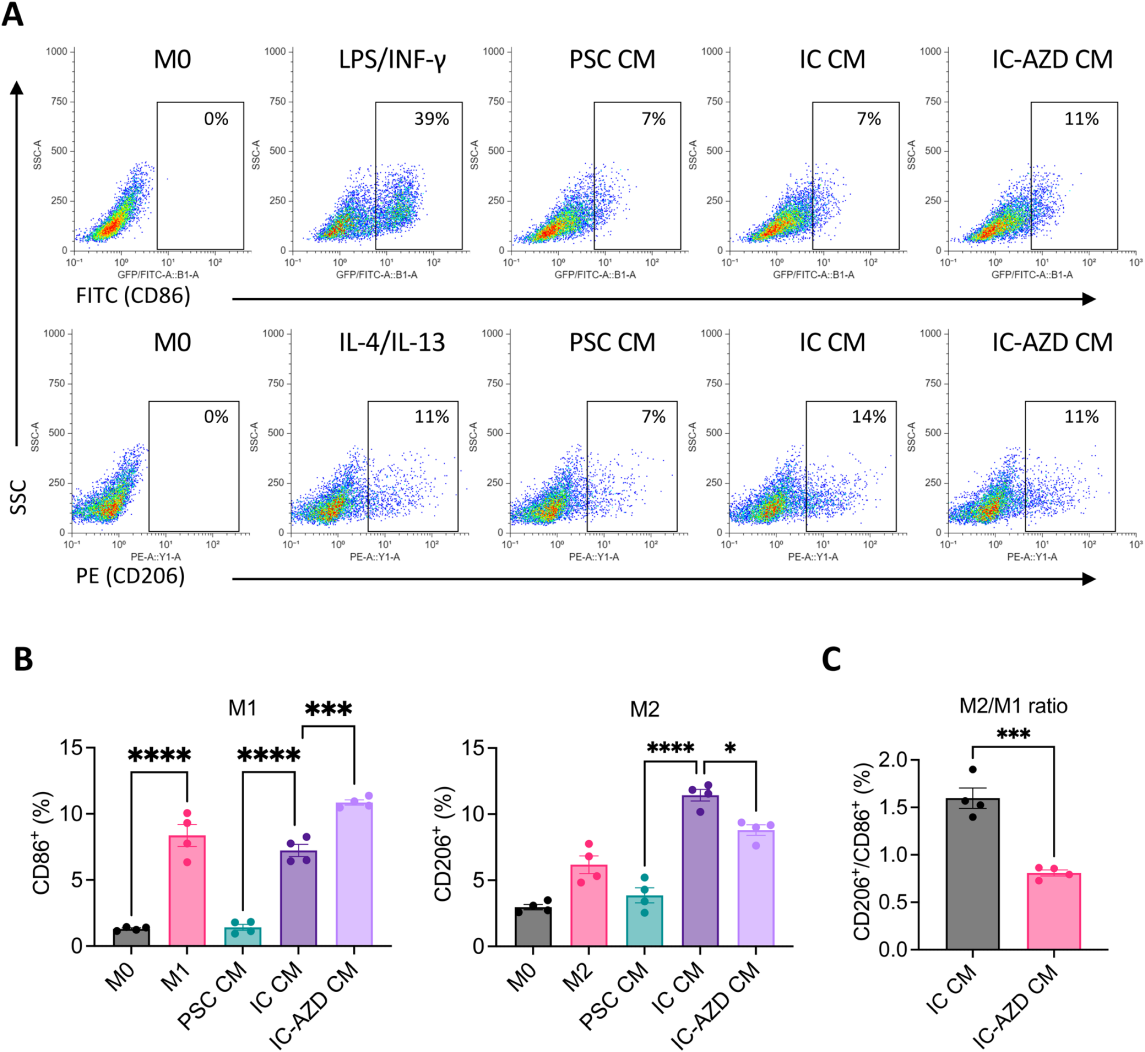
AZD inhibits iCAF‐mediated M2 macrophage polarization. (A) Representative flow cytometry dot plot panels and (B) analysis of THP‐1 differentiated macrophages stained for CD86 (M1 specific marker) and CD206 (M2 specific marker). (C) M2/M1 ratio analysis comparing IC CM and IC‐AZD CM‐treated macrophages. Graphical data represent mean ± SEM (*n* = 4). Statistical analysis represents One‐Way ANOVA (*p < 0.05, ***p < 0.001, ****p < 0.0001).

Studies have shown that iCAFs have a crosstalk with immune cells such as macrophages and have a strong role in controlling the tumor innate and adaptive immune system [29, 30]. Therefore, we investigated the effect of AZD on iCAF‐mediated macrophage polarization. We differentiated human THP‐1 monocytic cells using PMA, representing M0 macrophages, which were then exposed to LPS/IFNγ or IL4/IL13 to differentiate them into M1‐like or M2‐like macrophages, respectively. To study the effect of iCAF‐mediated effect on macrophage polarization, we treated M0 macrophages with CM collected from iCAFs or iCAF‐treated with AZD for 72 h. We then performed flow cytometry analysis to determine the effect on their polarization. Interestingly, M0 macrophages treated with iCAF CM displayed a notable upregulation of both CD86^+^ (M1‐like anti‐tumorigenic) and CD206^+^ (M2 type, pro‐tumorigenic) markers compared to the human PSC CM (Figure 4A,B). Importantly, macrophages exposed to AZD‐treated iCAF CM had an elevated expression of M1 macrophages and a reduced expression of the M2 marker when compared to the iCAF CM treatment (Figure 4A,B). The CD206^+^/CD86^+^ ratio clearly showed that AZD‐treated iCAF reduced M2‐like macrophages, indicating that treatment with AZD can suppress iCAF‐mediated activation of pro‐tumorigenic macrophages which leads to tumor immunosuppression (Figure 4C).

#### 3.4.1 AZD Inhibits Growth of 3D Stroma‐Rich Spheroids

**FIGURE 5:**
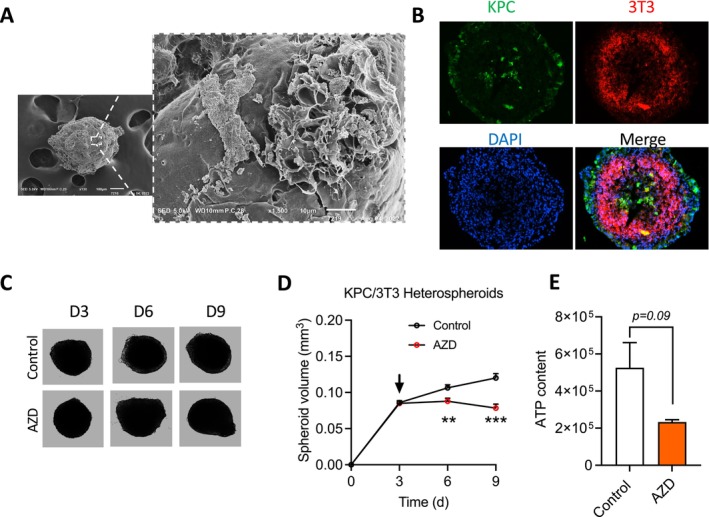
AZD inhibits KPC/NIH3T3 tumor spheroids. (A) SEM images of KPC/NIH3T3 tumor spheroid. Scale bar: 100 μm (low magnification), 10 μm (high magnification). (B) Fluorescence microscopic images of KPC/NIH3T3 spheroid cross‐section formed by cell tracker‐labeled KPC (green) and NIH3T3 (Red) on d3. Scale bar: 200 μm. (C) Representative microscopic images and volume analysis of spheroids growth assay. Scale bar: 500 μm. Bar graph is quantifying 9d timepoint (*n* = 9). (D) Quantification of CellTiter‐Glo 3D luminescence cell viability assay of spheroids growth (*n* = 3). Black arrow indicates onset of treatment. Graphical data represent mean ± SEM. Statistical analysis represents unpaired student *t*‐test (**p < 0.01, ***p < 0.001). (E) Representative microscopic images and volume analysis of human PANC‐1/PSC spheroids growth assay. Scale bar: 1000 μm. Black arrow indicates onset of treatment. Graphical data represent mean ± SEM (*n* = 6). Statistical analysis represents unpaired student *t*‐test.

To assess the impact of AZD on direct juxtracrine interactions between tumor and stroma, we employed a 3D stroma‐rich KPC/NIH3T3 spheroid model to effectively simulate the structural and functional characteristics of the stroma, enabling a comprehensive understanding of tumor‐stroma interactions as previously described in various cellular models [31, 32]. This model was initiated by co‐culturing KPC murine PDAC tumor cells with NIH3T3 murine fibroblasts in a 1:5 ratio. The NIH3T3 fibroblasts, upon interaction with KPC tumor cells, act as potential progenitors to CAFs, providing an enriched stromal component that closely mimics the TME. The resultant KPC/NIH3T3 heterospheroids exhibited a compact morphology, attributed to the contractile property of CAFs (Figure 5A). Conversely, when KPC cells were cultured alone, they failed to form cohesive spheroids, possibly due to the absence of the intercellular interactions which were offered by fibroblasts in heterospheroids. The surface topography of the KPC/NIH3T3 spheroids was visualized by scanning electron microscope (SEM) imaging and exhibited a non‐porous, compact structure, indicative of the profusely expressed ECM protein components secreted by the CAFs, resulting in a smooth spheroid surface (Figure 5A).

To further elucidate the spatial cellular arrangement within the spheroids, we implemented differential cellular labeling, wherein KPC and NIH3T3 cells were pre‐labeled with different fluorescent cell tracker dyes. Fluorescence microscopy images of the spheroid cross‐sections showcased a distinct distribution, where NIH3T3 cells predominantly occupied a circular band, lying between an outer thin layer of KPC cells and a core of majority of KPC cells (Figure 5B). Over a 9‐day culture, KPC/NIH3T3 spheroids displayed a consistent growth in volume. Interestingly, upon introduction of AZD on the third day, a > 30% decline in spheroid growth was observed (an average volume of 0.08 mm^3^ vs. 0.12 mm^3^ of the control group) on day 9 (Figure 5C). Moreover, CellTiter‐Glo 3D assay revealed reduction of lysed tumor spheroid ATP contents, as a marker for total spheroid cell viability (Figure 5D). Furthermore, to confirm the efficacy of AZD, we tested its effect on human pancreatic PANC‐1/PSC tumor 3D model, which showed similar growth inhibition trend over culture period of 9 days (Figure 5E).

### 3.5 AZD Attenuates the Tumor Growth and Liver Metastasis in KPC Tumor Model In Vivo

**FIGURE 6:**
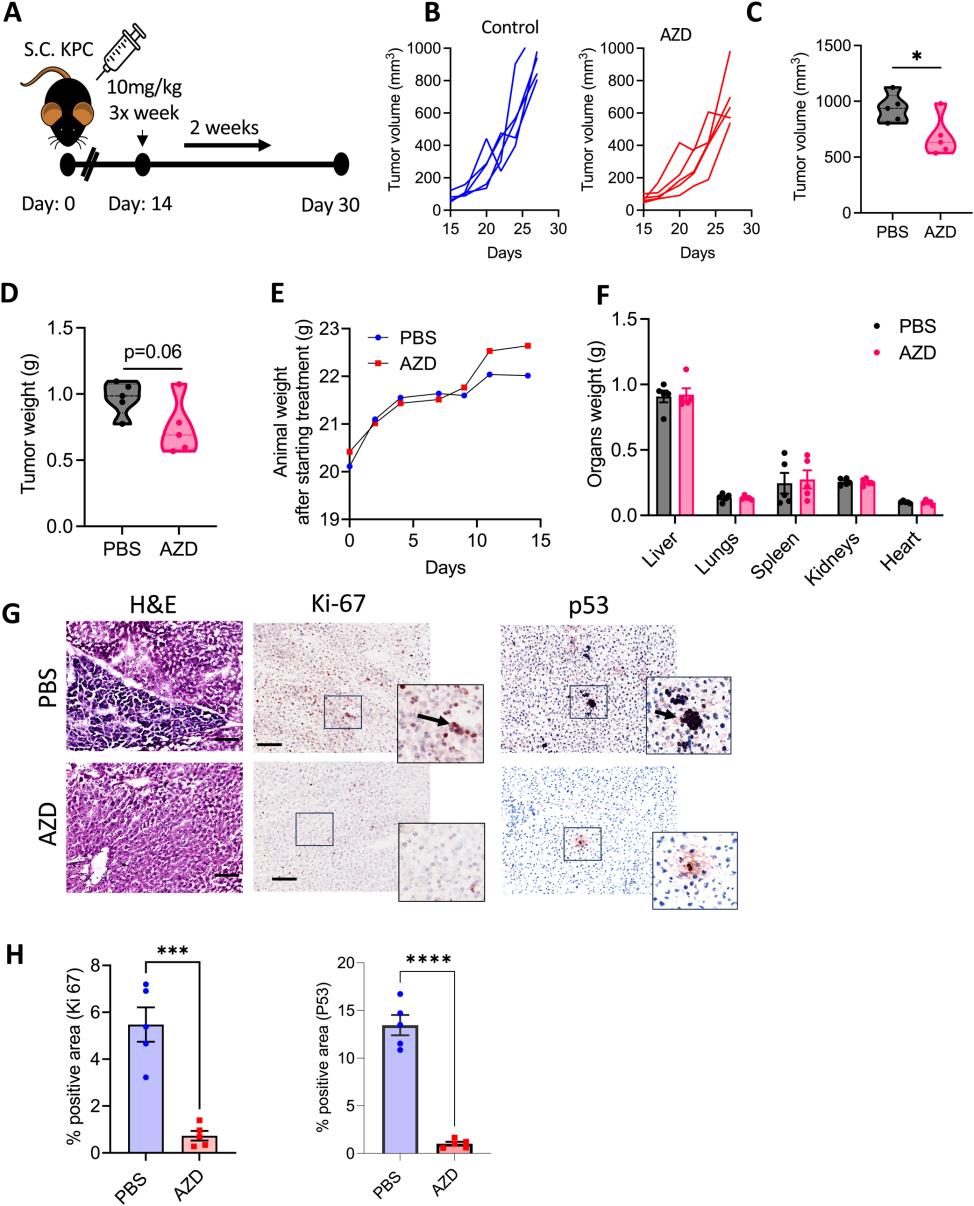
AZD4547 efficacy on KPC tumor growth in vivo. (A) Schematic illustration of the experimental setup of the animal experiment. (B) Tumor growth curve and (C) end tumor volume of individual subcutaneous KPC tumors. (D) Isolated tumor weight at the study endpoint. Graphical data represent mean ± SEM (*n* = 5). Statistical analysis represents a two‐tailed unpaired Student's *t*‐test. *p < 0.05. (E, F) Animal weight during the study and organ weights at the end of the study. (G) Representative immunohistochemical staining of isolated liver sections for hematoxylin and eosin (H&E) staining, the proliferation marker Ki‐67, and the tumor‐associated marker p53 on the liver sections. Scale bar: 100 μm (H) Quantification of Ki‐67—and p53‐positive areas. Data are presented as mean ± SEM (*n* = 5) and analyzed using a two‐tailed unpaired Student's *t*‐test (***p < 0.001, ****p < 0.0001).

To further assess the therapeutic potential of AZD, we utilized a heterotopically injected, syngeneic KPC PDAC murine tumor model. This model closely recapitulates PDAC features owing to its pertinent genetic alterations and tumor traits with abundant stroma and complete immune system [32, 33]. C57/BL mice were subcutaneously inoculated with KPC3‐Luc2 cells to establish tumors. Once tumors attained tumor volumes of 50–100 mm^3^ (about two weeks), mice were injected with AZD, i.p. thrice weekly (Figure 6A). Over the subsequent two weeks of therapeutic intervention, the AZD‐treated cohort demonstrated about a 20% reduction in tumor growth compared to the untreated mice (Figure 6B,C). In line with the tumor volume, at the termination of the study, a tumor weight reduction of about 20% was seen in the AZD‐treated group (Figure 6D). Additionally, the animals' whole body weight and isolated organ weights analysis showed no changes among both treatment groups, underscoring tolerable AZD dosing and suggesting no side effects manifested (Figure 6E,F).

Furthermore, we investigated the effect of AZD on liver metastasis by measuring bioluminescence in the isolated livers, facilitated by the luciferase‐expressing KPC3‐Luc2 cells. While there was a slight reduction in the bioluminescence signals in the AZD‐treated mice relative to the control group, it was significant due to the low signal obtained from the livers (Figure S4). No bioluminescence signal was detected in other organs among the PBS group (data not shown). To determine the metastatic dissemination, we next assessed the effect of AZD using histological analysis. Hematoxylin and eosin (H&E) staining was performed to compare liver tissue morphology between treatment groups. In the PBS control group, liver sections exhibited increased areas of large, hyperchromatic, and irregular nuclei, consistent with enhanced metastatic colonization. Remarkably, treatment with AZD resulted in a strong reduction of such areas (Figure 6G), indicating a significant attenuation of metastatic burden. To further validate these findings, immunohistochemical analysis was performed on isolated liver sections using the tumor‐associated marker p53 and the proliferation marker Ki‐67. Both markers were expressed in the PBS‐treated group, confirming the presence and proliferative activity of metastatic tumor cells within the liver (Figure 6G). Strikingly, AZD treatment resulted in a pronounced reduction in Ki‐67 and p53 staining intensity (Figure 6H), further supporting a decrease in metastatic colonization and tumor cell proliferation. Collectively, these results demonstrate that AZD significantly suppresses liver metastatic progression and reduces the establishment and proliferative capacity of metastatic tumor cells in the liver microenvironment.

### 3.6 Treatment With AZD Reduces iCAF and Reprogram M2‐TAMs In Vivo

**FIGURE 7:**
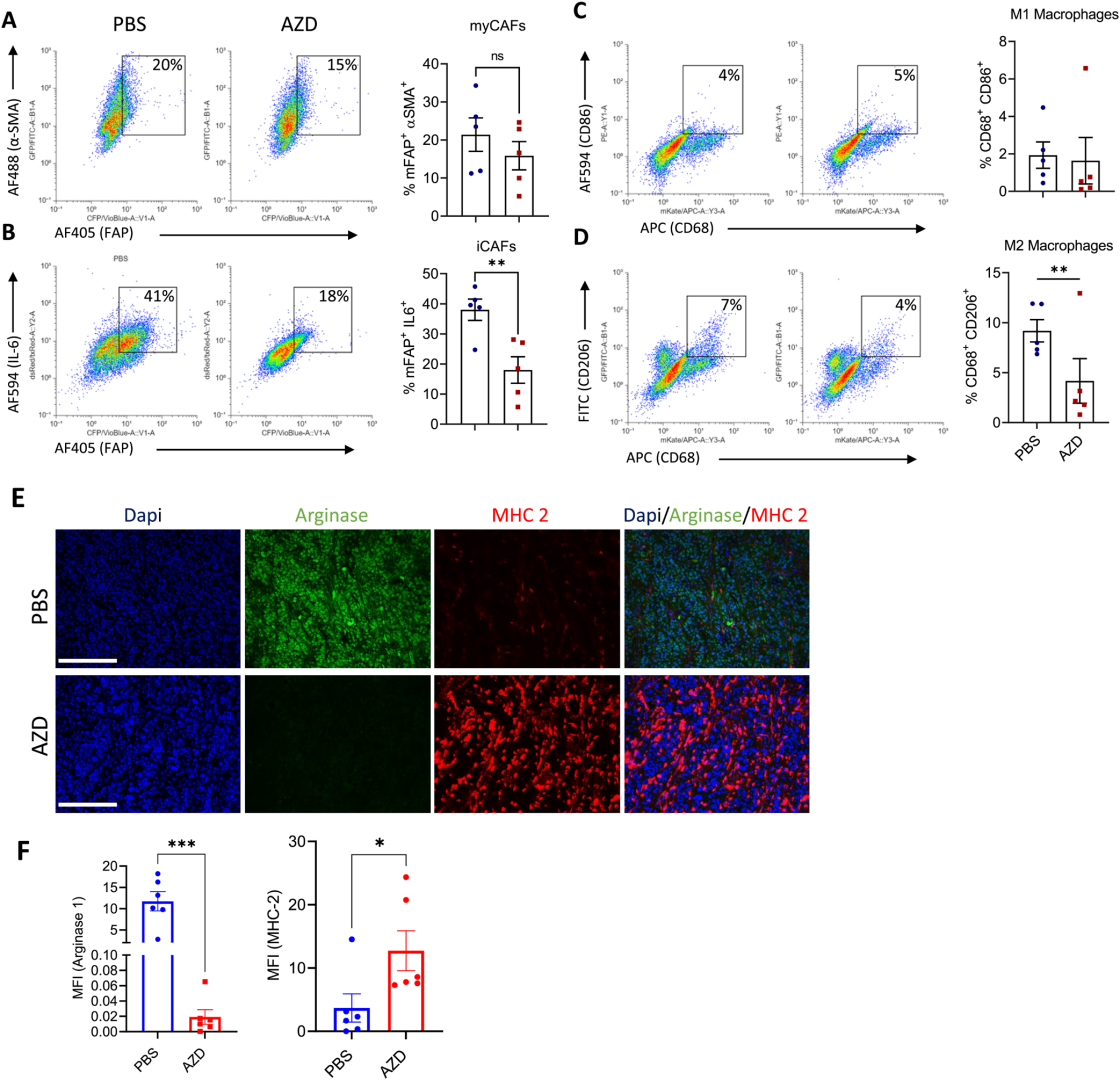
Effect of AZD on the stromal cell phenotype and macrophage polarization in vivo. (A–D) Flow cytometry dot plots and quantitative analysis depicting (A) FAP^+^ α‐SMA^+^ (myCAF) and (B) FAP^+^ IL‐6^+^ (iCAF), (C) CD68^+^ CD86^+^ (M1) and (D) CD68^+^ CD206^+^ (M2) stromal populations. Graphical data represent mean ± SEM (*n = 5*). Statistical analysis represents two‐tailed unpaired student *t*‐test. **p < 0.01. (E, F) Immunofluorescent staining of arginase (Arg1, M2 marker) and MHC‐II (M1 marker) on the histological tumor sections. Scale bar = 200 μm. *N* = 6. Statistical analysis represents two‐tailed unpaired student *t*‐test. *p < 0.05, ***p < 0.001.

To demonstrate the impact of FGFR inhibition on the phenotype of CAFs and TAMs, we isolated single cell suspensions from the isolated tumors and investigated cell‐specific markers for stromal cell populations (myCAFs, iCAFs, and TAMs) using flow cytometry. Our findings revealed that in the AZD‐treated group, the iCAFs (FAP^+^ IL‐6^+^) population was significantly reduced by > 50% compared to the PBS group, while the myCAF (FAP^+^ α‐SMA^+^) population was decreased slightly (Figure 7A,B). These data clearly indicate that inhibition of FGFR by AZD has a direct inhibitory effect on the iCAFs in vivo. Furthermore, interestingly, we found a notable decrease in the CD86^+^ CD206^+^ TAM population in the AZD‐treated group compared to the PBS group, while no noted effect was observed on CD68^+^ CD86^+^ M1 macrophages or on the M2/M1 ratio (Figure 7C,D).

Furthermore, we performed immunofluorescent staining on the tumor sections for M1 (MHC‐II) and M2 (Arginase) markers and found that AZD‐treated tumors had a reduction of MHC‐II and induction of Arginase (Figure 7E,F), confirming the flow cytometry data. These in vivo data are in line with our in vitro data in Figure 3, in which we showed that AZD‐treated iCAF CM reduces macrophage polarization to CD206^+^ M2 type. Furthermore, we examined the effect of AZD on the protein expression of collagen and vimentin in vivo using immunofluorescence analysis (Figure S5). We found that there was no effect on the collagen expression, while there was a slight trend of reduction in vimentin. This is in line with the flow cytometry data in which there was no effect on myCAFs, the responsible cell type for the ECM production.

## 4 Discussion

Next to previously proposed mechanisms related to cell survival and apoptotic pathways, this study innovatively demonstrates a novel mechanism of action of FGFR inhibitor AZD4547 in inhibiting iCAF‐induced tumor cell migration and M2 macrophage differentiation. The scRNAseq and our in vitro data show that FGFR1 is among the most overexpressed FGF receptors on iCAFs, and the treatment with AZD inhibited this overexpression significantly. AZD not only inhibited the activation of iCAF but also the induction of migration and EMT of PDAC cancer cells, shown with the conditioned media studies. Furthermore, AZD also inhibited iCAF‐induced macrophages' differentiation into M2 phenotype. Importantly, treatment with AZD reduced the 3D heterospheroid growth in vitro as well as in the syngeneic subcutaneous KPC murine tumor model in vivo. Furthermore, AZD–treated tumors showed a reduced iCAF population and M2‐type macrophages, as shown with flow cytometry analysis. Interestingly, there was a significant reduction in the liver metastasis in AZD‐treated mice which is in line with the inhibitory effect of AZD on iCAF‐induced tumor cell migration.

The role of CAFs in promoting EMT has been widely reported in various tumor types such as PDAC and esophageal squamous cell carcinoma [34, 35]. These functions are primarily exerted through interactions with tumor cells via cytokine secretion, ECM remodeling, and other paracrine mechanisms [36]. In view of iCAFs proteomic fingerprint, IL‐6 and CXCL12 arise as key factors promoting EMT functions within the TME [37, 38]. The signaling pathways activated by these molecules, such as JAK/STAT3 and CXCR4‐mediated pathways, result in the activation of transcription factors that induce EMT. This transition is marked by increased motility, invasiveness, and resistance to apoptosis, characteristics that are crucial for metastasis [37, 39, 40]. Notably, our results show that inhibition of FGFR by AZD leads to the inhibition of iCAF–induced paracrine signals on the tumor cells, leading to reduced their differentiation via EMT and migratory properties.

The correlation between the presence of CAFs and the recruitment of TAMs in the TME has been previously established in other cancers, such as colorectal cancer [41, 42]. The ability of CAFs to suppress anti‐tumor immunity has also been reported elsewhere [43, 44], which they exert directly via secretion of T‐cell suppressive factors or indirectly by stimulating M2 TAM activation and recruitment [45]. In particular, iCAFs have also been recently shown to significantly contribute to the immunosuppressive and pro‐tumorigenic TME [46]. In this study, we showed that AZD treatment led to a pronounced decrease in TAM polarization towards pro‐tumorigenic M2 phenotype in vitro as well as in vivo. These results also confirm the role of iCAFs in establishing the TAM dichotomy, thereby indirectly influencing tumoral progression and the immune landscape. The presence of both M1 and M2 markers upon exposure to iCAF CM reveals a complex interplay, potentially driving a mixed macrophage response. However, AZD treatment to iCAFs led to the shift in macrophage polarization to M2 type, evidenced by the ratio of CD206 (M2) and CD86 (M1) and immunofluorescent stainings for their markers (Figure 7), reinforcing our findings that targeted modulation of iCAFs reprograms the immunosuppressive into immune‐stimulating TME.

Furthermore, the inhibitory effect of AZD on the growth of 3D stroma‐rich spheroids and its attenuating impact on KPC tumor growth in vivo provide evidence of its potential to disrupt direct and paracrine interactions within the tumor stroma that are essential for tumor growth and metastasis. The marked reduction in intratumoral iCAFs and re‐polarization of TAMs upon AZD treatment highlight its impact on the reprogramming of the TME.

In conclusion, our study provides new evidence that therapeutic targeting of FGFR signaling in iCAFs leads to inhibitory effects on their pro‐tumorigenic and pro‐metastatic role via interruption of crosstalk with tumor cells and macrophages. This study sheds a new light on the role of FGFRs in tumor‐stroma crosstalk, in particular with iCAFs, and relevance to metastasis for which this inhibitor has been currently tested in clinical studies. One of the limitations of this study is the use of a subcutaneous PDAC model and further studies need to be performed in orthotopic or genetically engineered mouse models. Furthermore, extended studies are warranted to understand the effect on CAF‐driven resistance mechanisms against chemotherapy and immunotherapy.

## Author Contributions

Ahmed R. Mostafa and Ahmed G. Hemdan designed and performed experiments, collected data, analyzed them, and wrote the original manuscript. Franck Assayag performed in vivo experiments. Jai Prakash supervised the project, designed experiments, wrote, and edited the manuscript.

## Funding

This work was supported by EC | European Research Council (ERC) (Grant 101142157) and Egyptian Cultural and Educational Bureau (ECEB).

## Conflicts of Interest

The authors declare no conflicts of interest.

## Supporting information


**Figure S1:** (A) Publicly available scRNAseq data (analyzed using TISCH website) show the expression of myCAF and iCAF markers and FGFRs in different cell types (B) Effect of IL‐1α on the gene expression of α‐SMA (*ACTA2*) and *COL1A1* in human PSCs.
**Figure S2:** Effect of AZD on the growth of human PSC‐derived iCAFs. hPSCs were treated with IL‐1α with/without AZD at two different concentrations and Alamar blue assay was performed on *t* = 0 h (treatments) to *t* = 72 h. *n* = 3, Two‐way ANOVA, *p < 0.05.
**Figure S3:** Effect of AZD on human PSC‐derived myCAF phenotype markers. (A) Representative Immunofluorescence microscopic images and analysis of (B) α‐SMA and (C) Collagen 1. Scale bar: 200 μm. Graphical data represent mean ± SEM (*n* = 3). Statistical analysis represents One‐Way ANOVA. ***p < 0.001.
**Figure S4:** Bioluminescence signal analysis after injection of d‐luciferin at *t* = 10 min post‐injection.
**Table S1:** Primer sequences used for quantitative real‐time PCR (qRT‐PCR).
**Table S2:** Primary and secondary antibodies used for immunofluorescence staining.

## Data Availability

All data generated and analyzed during this study are provided within the manuscript and its Supporting Information. The representative images of the western blot, flow cytometry data, and microscopic images are included within the manuscript. Publicly available datasets were analyzed in this study through the following database available at DOI: 10.1038/s41422‐019‐0195‐y and analyzed using https://tisch.compbio.cn. Any additional data are also available from the corresponding authors on reasonable request.
