# Supplementary material for: FGFR Inhibitor AZD4547 Disrupts Inflammatory CAF Crosstalk With Cancer Cells and Macrophages and Attenuates Metastasis in Pancreatic Cancer
Source: FASEB J. 2026 Jul 2;40(13):e72101. doi: 10.1096/fj.202504573RR (PMC13329420; doi:10.1096/fj.202504573RR)
Supplement: Supplementary file 1 — Figure S1: (A) Publicly available scRNAseq data (analyzed using TISCH website) show the expression of myCAF and iCAF markers and FGFRs in different cell types (B) Effect of IL‐1α on the gene expression of α‐SMA (ACTA2) and COL1A1 in human PSCs. Figure S2: Effect of AZD on the growth of human PSC‐derived iCAFs. hPSCs were treated with IL‐1α with/without AZD at two different concentrations and Alamar blue assay was performed on t = 0 h (treatments) to t = 72 h. n = 3, Two‐way ANOVA, *p < 0.05. Figure S3: Effect of AZD on human PSC‐derived myCAF phenotype markers. (A) Representative Immunofluorescence microscopic images and analysis of (B) α‐SMA and (C) Collagen 1. Scale bar: 200 μm. Graphical data represent mean ± SEM (n = 3). Statistical analysis represents One‐Way ANOVA. ***p < 0.001. Figure S4: Bioluminescence signal analysis after injection of d‐luciferin at t = 10 min post‐injection. Table S1: Primer sequences used for quantitative real‐time PCR (qRT‐PCR). Table S2: Primary and secondary antibodies used for immunofluorescence staining. [file FSB2-40-e72101-s001.pdf]

FGFR inhibitor AZD4547 disrupts inflammatory CAF crosstalk with cancer cells and macrophages and attenuates metastasis in pancreatic cancer

Ahmed M.R.H. Mostafa[a,#], Ahmed G. Hemdan[a,b,c,#], Franck Assayag[a], Jai Prakash[a,b,*].

[a]Engineered Therapeutics, Department of Bioengineering Technology, University of Twente, Enschede, The Netherlands.

[b]Department of Medical Biosciences, Radboud University Medical Centre, Nijmegen, The Netherlands.

[c]Department of Pharmacology and Toxicology, Faculty of Pharmacy, Assiut University, Assiut, Egypt.

[#] Equal contribution and shared authorship

* Corresponding author

Department of Medical BioSciences (MBS)

Radboud University Medical Centre

Geert Grooteplein Zuid 10

6525 GA, Nijmegen

The Netherlands

Email: jai.prakash@radboudumc.nl

Supplementary materials

Table S1. Primer sequences used for quantitative real-time PCR (qRT-PCR).

Gene	Forward primer	Reverse primer
FGFR1(b)	AGAGCGGGGAGTATGTGTGTAA	CCTCTCTTCCAGGGCTTTTGC
FGFR2 (b)	ACAGCTTCCCCAGACTACCT	CAGGGGGATACGTTTGGTCA
FGFR3 (b)	CGACGAGTACCTGGACCTGT	CCTCACATTGTTGGGGACCA
FGFR4	AGTTCTGCCTACAGGACACG	ACAGGAGTCCCACCGTGTAT
RPS18	TGAGGTGGAACGTGTGATCA	CCTCTATGGGCCCGAATCTT
CXCL12	TGCCCTTCAGATTGTAGCCC	GCGTCTGACCCTCTCACATC
CSF3	TAGCGGCCTTTTCCTCTACC	CAGTTCTTCCATCTGCTGCC
LIF	CTGTCAACTCCTGGGGAAGT	ACATCTGGACCCAACTCCTG
CXCL1	ATGCCAGCCACTGTGATAGA	TCCCCTGCCTTCACAATGAT
ACTA2	CCCCATCTATGAGGGCTATG	CAGTGGCCATCTCATTTTCA
COL1A1	GTACTGGATTGACCCCAACC	CGCCATACTCGAACTGGAAT

Table S2. Primary and secondary antibodies used for immunofluorescence staining.

**Antibody**	**Source**	**Dilution**
IL-6 Polyclonal antibody (P620)	Thermo Fisher Scientific	1:100
SNAIL Monoclonal Antibody (MA5-14801)	Thermo Fisher Scientific	1:250
SNAI2/SLUG polyclonal antibody (12129-1-AP)	Proteintech	1:400
FGF Receptor 1 rabbit monoclonal antibody (D8E4)	Cell Signaling	1:200
Anti- α-SMA monoclonal antibody (A2547)	Thermo Fisher Scientific	1:500
Goat anti-collagen type 1 collagen antibody (1310-01)	Southern Biotech	1:250
MHC II Monoclonal Antibody (14-5321-82)	Thermo Fisher Scientific	1:300
Arginase I Antibody (SC-20150)	Santa Cruz	1:200
Alexa Fluor™ 488 donkey anti-rabbit (A-21206)	Thermo Fisher Scientific	1:100
Alexa Fluor™ 594 donkey anti-rabbit (A-21207)	Thermo Fisher Scientific	1:100
Alexa Fluor™ 594 donkey anti-goat (A-11058)	Thermo Fisher Scientific	1:100
Alexa Fluor™ 488 donkey anti-mouse (A-21202)	Thermo Fisher Scientific	1:100
Alexa Fluor™ 594 donkey anti-rat (A-11007)	Thermo Fisher Scientific	1:100

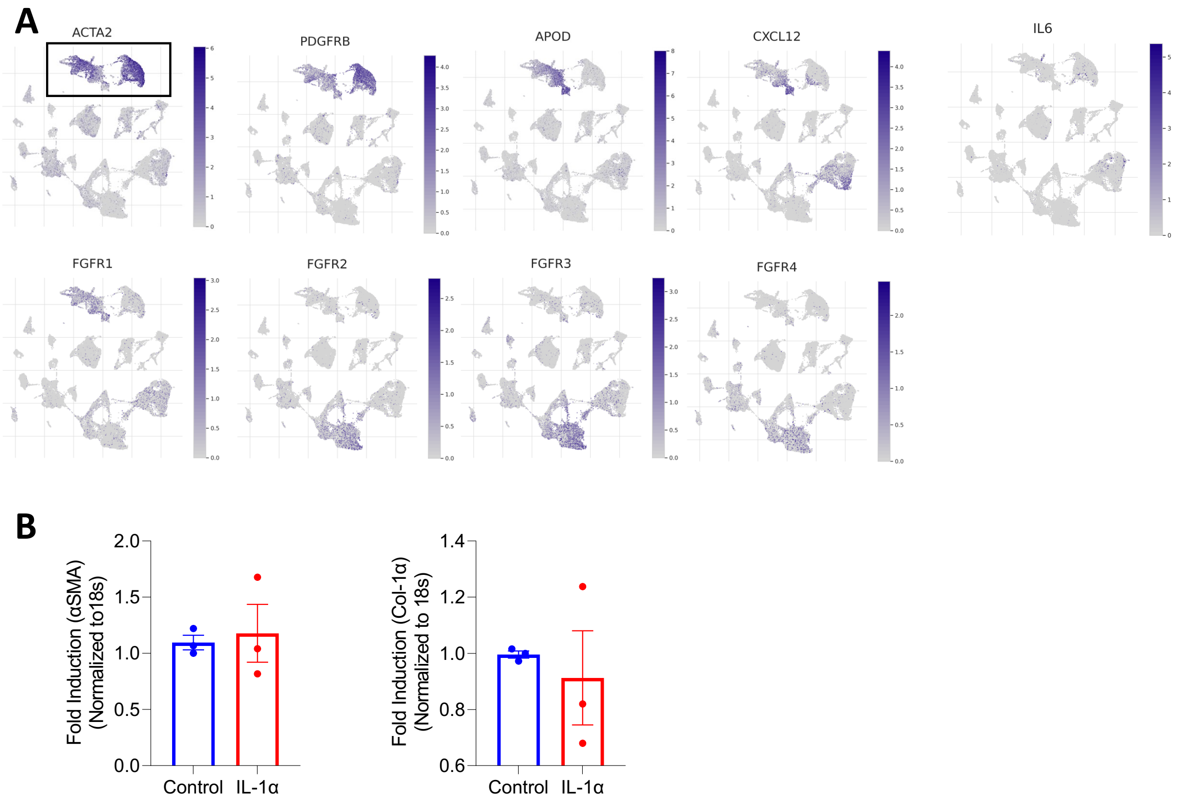

Figure S1. (A) Publicly available scRNAseq data (analyzed using TISCH website) show the expression of myCAF and iCAF markers and FGFRs in different cell types (B) Effect of IL -1α on the gene expression of α-SMA (*ACTA2*) and *COL1A1* in human PSCs.

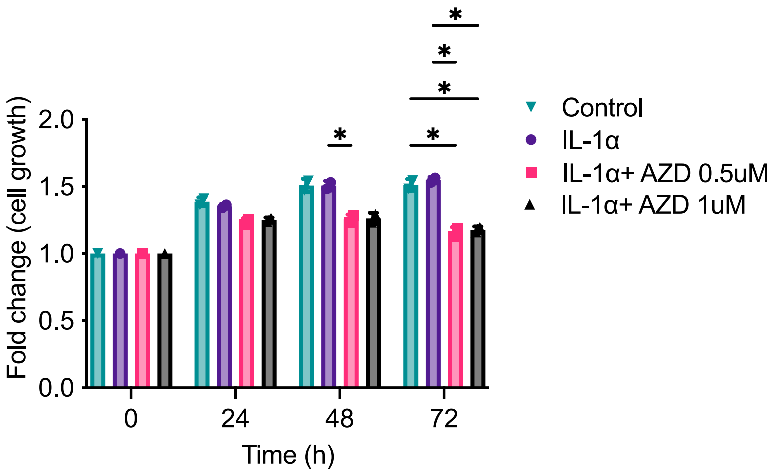

Figure S2. Effect of AZD on the growth of human PSC-derived iCAFs. hPSCs were treated with IL-1α with/without AZD at two different concentrations and Alamar blue assay was performed on t=0h (treatments) to t=72h. n=3, Two-way ANOVA, *$p < 0.05$.

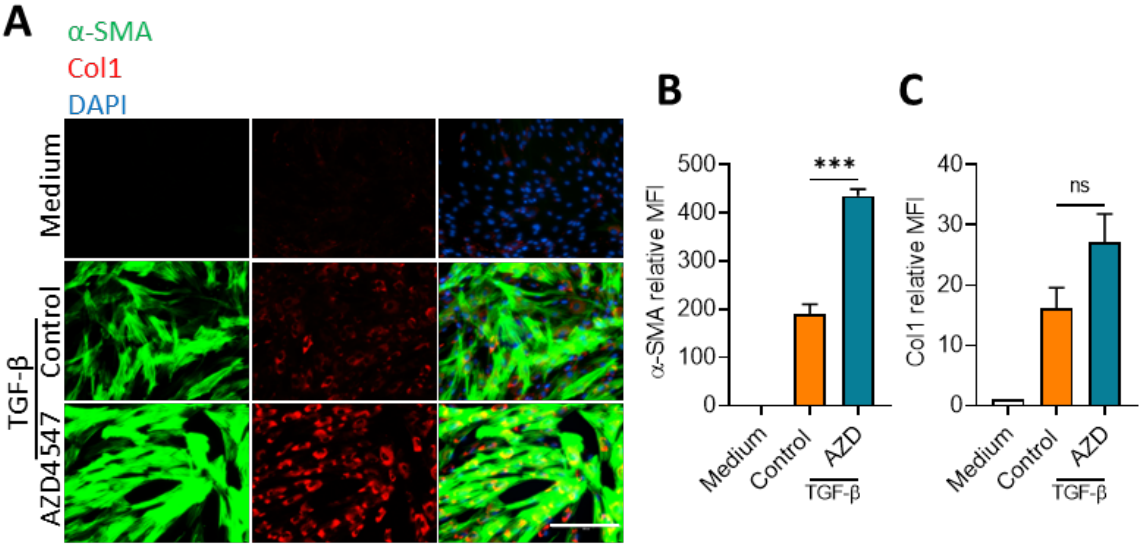

Figure S3. Effect of AZD on human PSC-derived myCAF phenotype markers. (A) Representative Immunofluorescence microscopic images and analysis of (B) α-SMA and (C) Collagen 1. Scale bar: 200 μm. Graphical data represent mean ±SEM (n=3). Statistical analysis represents One-Way ANOVA. ***$p < 0.001$.

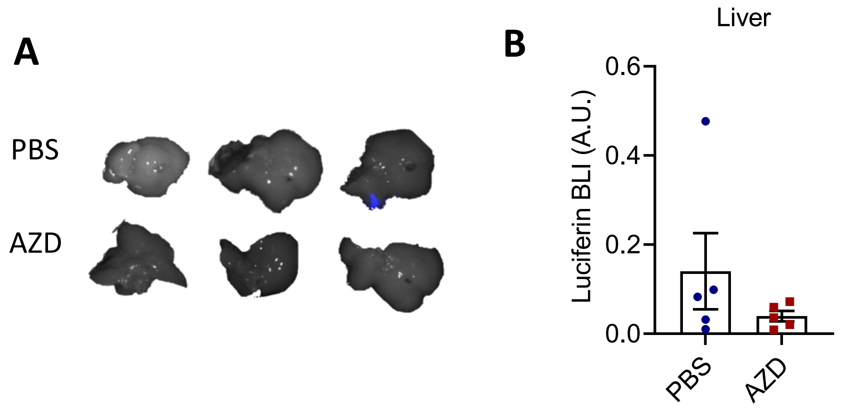

Figure S4. Bioluminescence signal analysis after injection of D-luciferin at t=10 min post-injection.
